# Recent Advances in Sensing Oropharyngeal Swallowing Function in Japan

**DOI:** 10.3390/s100100176

**Published:** 2009-12-28

**Authors:** Takahiro Ono, Kazuhiro Hori, Yuji Masuda, Toyohiko Hayashi

**Affiliations:** 1 Division of Oromaxillofacial Regeneration, Osaka University Graduate School of Dentistry, 1-8 Yamada-oka, Suita, Osaka 565-0871, Japan; 2 Division of Dysphagia Rhabilitation, Niigata University Graduate School of Medical and Dental Sciences, 2-5274 Gakkocho-dori, Niigata 951-8514, Japan; E-Mail: hori@dent.niigata-u.ac.jp; 3 Graduate School of Oral medicine, Matsumoto Dental University, 1780 Gohara, Hirooka, Shiojiri, Nagano 399-0781, Japan; E-Mail: masuday@po.mdu.ac.jp; 4 Department of Biocybernetics, Faculty of Engineering, Niigata University, 8050 Ninomachi, Igarashi, Nishi-ku, Niigata 950-2181, Japan; E-Mail: hayashi@eng.niigata-u.ac.jp

**Keywords:** swallowing, dysphagia, lip, tongue, pharynx, larynx, biomechanical sensing, rehabilitation medicine

## Abstract

Dysphagia (difficulty in swallowing) is an important issue in the elderly because it causes aspiration pneumonia, which is the second largest cause of death in this group. It also causes decline in activities of daily living and quality of life. The oral phase of swallowing has been neglected, despite its importance in the evaluation of dysphagia, because adequate protocols and measuring devices are unavailable. However, recent advances in sensor technology have enabled straightforward, non-invasive measurement of the movement of important swallowing-related organs such as the lips and tongue, as well as the larynx. In this article, we report the present state and possibility of clinical application of such systems developed in Japan.

## 1. Introduction

Dysphagia, a disease of the feeding mechanism in humans, has become a matter of increasing concern with the aging of the global population over the last 20 years of the 20th century. Because of aging and various diseases of later life, such as sensori-motor disorders caused by cerebrovascular disease or neurological disease, the number of elderly patients with dysphagia has risen, thus promoting studies in the fields of medicine, dentistry, and engineering as social concern has increased. Recent USA estimates of the morbidity rate are 15–40% in those aged 60 years or older, amounting to approximately 6,200,000 persons [1]. Dysphagia can not only prolong hospital stays and increase medical costs, but also lead to delayed rehabilitation due to malnutrition and decreased quality of life, eventually increasing the personal and financial burdens on nursing care and social welfare services.

Swallowing is a series of processes that involve an array of organs. First, a bite of food taken into the mouth through the lips is processed to a bolus by masticatory jaw movement for easy swallowing; the swallowing reflex then occurs, transferring the bolus down the throat into the esophagus, before peristaltic waves finally carry it from the esophagus to the stomach. The movement of each organ proceeds in some sort of coordinated manner under the control of a neuromuscular mechanism. If this coordinated movement is disrupted, a residue of bolus may occur in the oropharynx, or early invasion, leading to life-threatening aspiration pneumonia due to inhalation (aspiration). To date, the diagnosis of dysphagia has been made with videofluorography (VF). VF can visualize the individual movements of the organs and locate a bolus during the entire process of swallowing, but cannot quantitatively assess the biomechanical effect of each movement. The examination is also limited because it requires technical experts and large equipment, and frequent examination is not allowed because of the possible adverse effects of radiation exposure. In these contexts, the development of a non-invasive, quantitative method of assessing movement in swallow-related organs is currently one of the most important issues in the treatment and rehabilitation of dysphagia [2].

This research subject has been of interest to both clinicians and researchers in Japan for many years. In the field of dentistry in particular, which has had a long association with engineering fields in the analysis of jaw movement during mastication, a wide variety of studies regarding oropharyngeal motor sensing have been performed over the past 10 years, with some devices having been developed for clinical use. All of the sensing systems follow conventional methods in principle; however, different techniques and designs have been tested specifically to perform non-invasive measurement of morphological changes in the luminal oropharynx and to extract biomechanically relevant indicators from recorded data. This review article aims to provide an overview of the representative sensing systems developed in Japan for measuring oropharyngeal movement during swallowing.

## 2. Outline of Biomechanical Assessment of Swallowing-Related Organs

**Figure 1.**
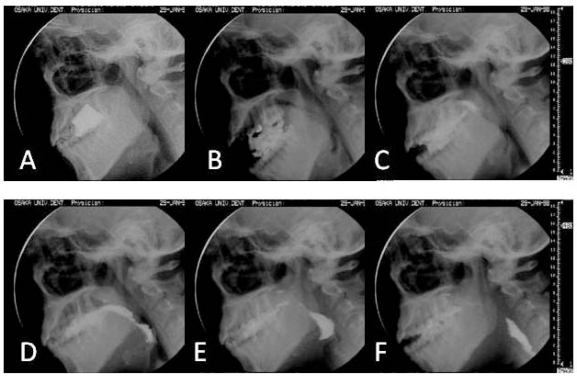
Process of mastication and swallowing of gummy jelly observed by VF [3]. A: before mastication, B: comminution by masticatory jaw movement (processing), C: bolus formation between the dorsum of the tongue and the soft palate, D: bolus transport and aggregation in the epiglottic valleculae, E: propulsion of the bolus into the pharynx and rotation of the epiglottis (elevation of the soft palate, tongue, hyoid bone, and larynx by the swallowing reflex), F: hypopharyngeal transit of the bolus following contraction of the pharynx.

VF imaging (Figure 1) is currently the best way of evaluating the swallowing function because it enables visualization of the movement of all anatomical components relating to chewing and swallowing [3]. These components include the lips, cheeks, jaw, tongue, hyoid bone, pharynx, larynx, and esophagus. VF imaging also enables visualization of the passage of a food or drink containing contrast medium in two dimensions (sagittal and frontal). The application of this imaging, however, involves radiation exposure, and is therefore limited to patients with severe dysfunction in chewing and swallowing.

A series of swallowing processes, from oral ingestion of food, transfer through the pharynx and esophagus, and delivery to the stomach, can be shown using the VF-based sequential model. The distinction between normal and abnormal presentations has also been described using this swallowing model [3–7]. Notable findings were reported by Palmer and Hiiemae [6], who established a process model of swallowing by specifically analyzing solid food ingestion, transport of food from the front to the molar teeth (Stage I transport), comminution and bolus processing by mastication (processing, Figures 1B,C), bolus transport from the oral cavity to the pharynx (Stage II transport, Figure 1D), and swallowing reflex and passage through the hypopharynx (hypo-pharyngeal transit, Figure 1E,F). They explained the events that occur at each stage of the swallowing process (Table 1) and defined their physiological significance [7]. This model has been widely accepted and is now used as the standard in the diagnosis of dysphagia as well as for understanding the normal swallowing process.

Based on the concept of the Process Model, we describe novel sensing methods developed after 1998 in Japan for monitoring the movement of the three organs involved in swallowing: lip movement critical to food ingestion; tongue movement critical to food transport in the oral cavity, comminution and bolus formation, and bolus transport to the pharynx; and finally, laryngeal movement critical to bolus passage through the pharynx. Although jaw, pharyngeal and esophageal movements are also integral for swallowing, in this review, sensing techniques such as jaw tracking systems and manometry are referred to only in terms of their relationship to novel techniques. Electromyography has been also referenced in this manner because it is not a novel technique, having been described in numerous research articles for evaluation of the activity of swallowing-related muscles.

## 3. Sensing of Lip Movement

### 3.1. Lip Movement in Swallowing

Along with the jaw, the upper and lower lips open and close the mouth during swallowing, in motion that largely depends on the contraction and relaxation of the orbicular muscle of the mouth. When food is ingested, the lips are open, the jaw opens to the size of the food, and the food is held by the incisors. During mastication, the lip muscles contract in the opening phase while relaxing in the closing phase, in coordination with the masticatory movement of the jaw. It is well known that lip closure occurs in response to the swallowing reflex because unless the lips are closed at the time of hypopharyngeal transit, a lack of pressure formation within the oropharynx prevents the food bolus from being easily swallowed. The role of lip closure is highly important with regard to ingestion of food into the oral cavity, mastication, and swallowing of the bolus. It is reported that lip function declines in older people [8]; however, there is currently no established method for the assessment of lip function.

### 3.2. Assessment of Lip-Closing Force

Lip-closing force is measured as maximal voluntary lip-closing force or functional lip force. Maximal voluntary lip-closing force increases from infancy through childhood in healthy subjects [9–11], and shows a remarkable increase at 10–11 years of age. In contrast, it has been suggested that age-related changes are not observed in those 60 years or older [12] and that lip-closing force decreases in the frail elderly with increasing age [13]. Some reports suggest that lip-closing force may be affected by dental occlusion and maxillofacial morphology [14–17], or the morphological characteristics of the soft tissues [18–20]. Lip-closing force in childhood is known to be closely related to masticatory performance from studies that investigated its relationship with other functions [21]. Maximal voluntary lip-closing force, commonly assessed by the measurement value of lip pressure imposed on the oral vestibule, can be improved by myofunctional therapy of the mouth, which has been proven effective for strengthening lip force [22,23].

Functional lip-closing force is measured during swallowing or ingestion. Subjects hold a piece of material with a strain gauge, manufactured as thin as possible, between the lips during measurement. A previous study reported no difference between adults and the elderly regarding lip pressure during swallowing [24]. As for maximal lip-closing force, myofunctional therapy can enhance lip force during swallowing [23], suggesting that an increase in muscle strength of the lips may have an influence on functional closing force. Lip force at food intake is measured using a small plate containing a strain gauge on which food is placed; this is used as an indicator of ingestion force. Lip-closing force at the time of ingestion varies according to the type of food. In addition to these two types of lip force, the results of a previous study that measured voluntary control of lip-closing force using a special device suggested that delicate control of lip-closing force can be difficult in patients who have central nervous system disorder following brain injury [25].

**Figure 3.**
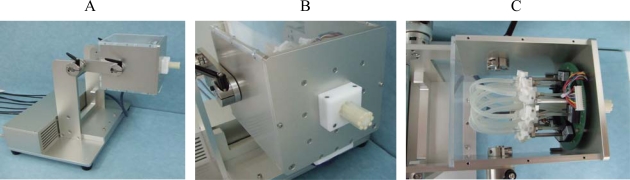
Apparatus for measuring multidirectional lip-closing force using air pressure sensors. A: overview, B: probe, C: inside of the sensing box. Changes in air pressure detected in the probe are measured by eight pressure sensors located in the sensing box.

Two simple devices are currently available to measure maximal voluntary lip-closing force: LIP DE CUM® (Cosmo Instruments, Hachiouji, Tokyo, Japan, Figure 3A) and Beauty Health Checker® (Patakara, Musashino, Tokyo, Japan, Figure 3B). In both devices, the sensor is held between the upper and lower lips to assess the maximal force produced to close the lips. LIP DE CUM is used with a dedicated lip holder (Ducklings®), while the Beauty Health Checker® records maximal force by placing the covered sensor in direct contact with the lips. These devices can only record forces that act in the vertical direction.

### 3.3. Novel Equipment: A System for Measuring Multi-Directional Lip-Closing Force

Movement of the lips is generated by the orbicularis oris muscle and other related facial muscles that make various other complex movements. The main muscle responsible for lip closure movement is the orbicularis oris, along with other facial muscles. Several studies that separately recorded the force of the upper and lower lips suggested that the lower lip was stronger than the upper in maximal voluntary lip-closing [26]; that the lower lip was more deft than the upper at controlling force, as confirmed by tasks requiring a certain level of force [27]; and that the lower lip showed a more stable response to tactile stimulation around the lips [28]. It is therefore considered that movement control differs between the upper and lower lips. When these findings are applied to lip-closing force, the force could be specific to the direction of lip-closing force; e.g., upward and downward, or left and right directions. Masuda *et al.*, [29] were the first to develop multidirectional lip-closing force measurement systems capable of measuring lip force from eight directions, thus enabling detailed analysis of lip function. Two such systems are discussed in this review.

The first system determines lip force by detecting pressure variations within a silicon tube, so that the contact surface with the device has as little effect as possible on measurement in detecting subtle force exerted by the soft lip tissue (Figure 3A). The device has a probe containing eight silicon tubes with closed tips that are placed in a circle to surround a plastic core (Figure 3B). Each tube is connected to a pressure transducer (PA-100, COPAL ELECTRONICS, Figure 3C). The lips are closed around the probe, and lip force imposed on the tubes is recorded as a change in air pressure.

**Figure 4.**
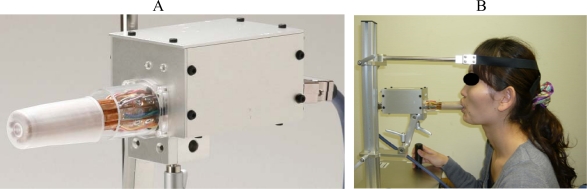
Apparatus for measurement of multidirectional lip-closing force using strain gauges. A: probe, B: patient set-up. The subject’s head is fixed, with Camper’s line parallel to the probe. The subject is instructed to close the lips with maximum effort.

Masuda then developed another type of apparatus using a strain gauge (Figure 4). Its probe (Figure 4A) is made of eight phosphor-bronze plates (5 mm in width, 2 mm thick, length of free unit 100 mm), each with a strain gauge (KFG-2-12, Kyowa Electronic Instruments). These plates are mounted on a plastic surface in the shape of a regular octagon and can determine multidirectional forces.

Because both of these devices are stereotactic, the subject’s head is positioned with the Camper’s line parallel to the horizontally adjusted probe; to record maximal voluntary lip-closing force, the subject places the probe in their mouth and closes their lips (Figure 4B).

**Figure 5.**
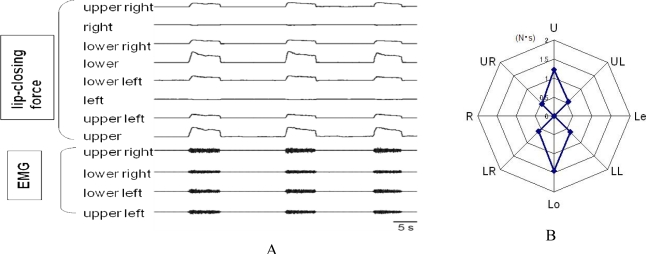
Example of the recording of multidirectional lip-closing force. A: lip-closing force in 8 directions and EMGs recorded from four lip muscle regions during maximal lip closing. B: radar chart showing the impulses of lip-closing force. The magnitude of the force differs among the eight directions.

**Figure 6.**
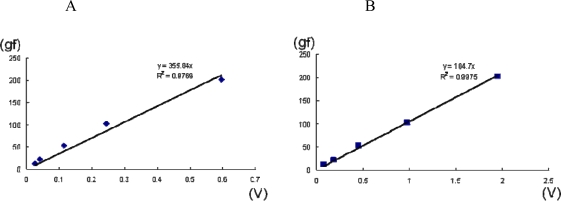

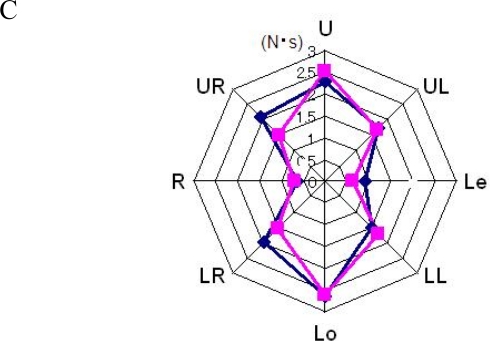
Comparison of the two types of apparatus that Masuda *et al.*, developed. A, B: Calibration lines for the measurement of multidirectional lip-closing force for devices using air-pressure sensors and strain gauges, respectively. The output of each apparatus is represented by voltage (V) in the abscissa and force (gram-force (gf)) in the ordinate. C: Radar chart of the impulses of lip-closing force in one subject. The blue and pink lines indicate multidirectional lip closing, as measured by devices using air pressure sensors and strain gauges, respectively.

Figure 5 shows the forces arising from eight different directions (*i.e.*, multidirectional lip-closing forces), as recorded by the strain gauge apparatus. The lip-closing forces shown are derived from eight directions: the upper, lower, right, and left sides of the lips and four additional oblique directions between these four directions (Figure 5A). A radar chart of the maximal voluntary lip-closing force is shown for each direction in Figure 5B. This illustrative approach helps us understand the directional characteristics of lip-closing force. Comparing the two types of system on the basis of calibration logs, accuracy is higher for the strain gauge apparatus than for the air pressure apparatus, even though both are highly accurate (Figure 6A, B). In the same subject, however, the multidirectional lip-closing force recorded by the two apparatuses was similar (Figure 6C), suggesting that the force balance from each direction reflects the characteristics of the subject rather than apparatus performance.

Measurement of multidirectional lip-closing force can be used to quantify variable forces in the balance ratio between up and down or left or right. There is currently no available equipment that can detect the balance ratio of the lip force in terms of motor function of the oropharynx. Development of such an apparatus would enable clinicians to diagnose labial movement disorder with high sensitivity by assessing maximal voluntary lip-closing force in absolute values, as well as directional force balance.

## 4. Sensing of Tongue Movement

### 4.1. Tongue Movement in Swallowing

As described in the process model (Table 1), tongue movement is varied and active throughout the entire swallowing process. In the ingestion stage, the tongue is positioned low in the mouth to make space for the food. In Stage I transport, the tongue then collects a bite of food and transfers it posteriorly, placing it on the molar occlusal surfaces by rotational movement. During processing, the tongue lifts up in the closing phase of mastication and repeatedly replaces food particles that were not masticated in the opening phase back onto the occlusal surfaces. Tongue movement in the oral cavity is three-dimensional during this period and highly skillful. The food bolus formed in the course of processing is slowly carried into the pharynx by the backward and forward movement of the tongue, activated in Stage II transport, to wait for the swallowing reflex. At the moment that the swallowing reflex occurs, the tongue root shifts backward at almost the same time as lifting up strongly to induce a pressure gradient that propels the bolus to the esophagus, in tandem with pharyngeal constriction and relaxation of the upper esophageal sphincter opening.

It is impossible to quantitatively sense and analyze all of these tongue movements. In the 1990s, however, VF and manometry were introduced simultaneously to monitor pressure flow dynamics in the oropharynx during swallowing. These methods demonstrated that pressure produced by contact between the tongue and palate is the most significant driving force in bolus transportation [30,31] and that the tongue controls the contact pressure with the food bolus in response to its volume and texture [32–34]. These findings in turn led to the development of devices for sensing the pressure produced between the tongue and palate. Several commercially available sensing devices were developed in the USA in the mid-1990s, such as the Iowa Oral Performance Instrument (IOPI, Blaise Medical, Hendersonville, TN, USA), incorporating a single bulb-shaped pressure sensor [34], and Air Filled Bulbs (Kaypentax, Lincoln Park, NJ, USA) with three sensing points [35]. These devices have been applied in evaluating maximal tongue pressure and intra-oral swallowing pressure.

### 4.2. Sensing Probe

**Figure 7.**
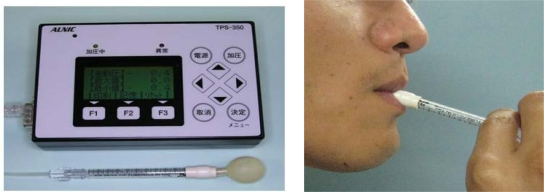
Disposable balloon probe system (photos courtesy of Dr. Kazuhiro Tsuga and Prof. Yasumasa Akagawa, Hiroshima University Graduate School of Medicine, Dentistry, and Pharmacy).

A novel tongue pressure gauge with a sensing probe was developed in 2002 by Hayashi *et al.*, (Handy Probe, Figure 7) [36]. Contact pressure in the tongue, detected by a small balloon-like sensing bulb at the end of a syringe inserted into the oral cavity, is conveyed to the pressure transducer of the main unit through the air contained in the tube, and the magnitude of pressure appears on the display. This device showed favorable performance in large studies because of its simplicity of use. A previous study determined age-related decrease in maximal tongue pressure based on the maximal voluntary isometric contraction measured in Japanese subjects of different age groups [37]. It is also reported that decreased maximal voluntarily isometric contraction in older subjects appears to reflect clinical signs of dysphagia [38]: as high maximal voluntary isometric contraction increases, the food residue observed in the oral cavity shows a decrease after masticating at a certain number of times and swallowing once, in the aged [39]. Following extensive research, this device has been developed for clinical use and is expected to be commercially available in the near future.

### 4.3. Artificial Palate with Sensors

**Figure 8.**
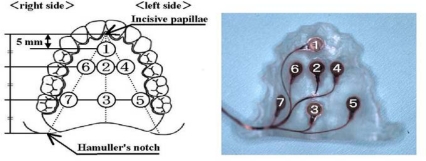
Location of pressure sensors (left) and an artificial palate (right). Each sensor is located with reference to the anatomical landmarks of the maxilla, such as the incisive papillae and Hamuller’s notch [47].

**Figure 9.**
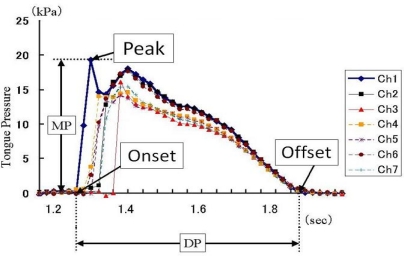
Example of the recording and method of analysis of tongue pressure production (MP, magnitude of tongue pressure; DP, duration of tongue pressure) [47].

Dentists are highly aware of the effect of deformation of the maxillary denture palate on problems with articulation, mastication, and swallowing. The importance of contact between the tongue and palate for normal function of the tongue can be understood from the efficacy of palatal augmentation prosthetics in the rehabilitation of post-glossectomy patients. On the basis of this previous clinical experience, there have been many attempts in Japan since the late 1990s to measure tongue pressure using pressure sensors inserted in palatal plates or maxillary dentures [40–46]. Ono *et al.*, referring to previous studies, recorded the waveform of tongue pressure at different sites of the hard palate using an artificial palate implanted with seven pressure sensors (Figure 8) to reveal the basic pattern of tongue pressure during swallowing of water (Figure 9) [47].

The pressure pattern of the tongue rising against the palate during swallowing is characterized by the order of onset, duration, and the maximal magnitude of tongue pressure at each site [47]. When 15 mL of water was ingested, tongue pressure occurred first in the antero-median region (Ch. 1), followed by the antero-circumferential region (Chs. 4 and 6). On the midline, tongue pressure was generated in the order Ch. 1, Ch. 2, and Ch. 3, from anterior to posterior; Ch.3 was the last among the seven sensors. Tongue pressure attained at the different sites reached a peak immediately after generation and fell simultaneously, declining slowly. Values of duration and maximal magnitude of tongue pressure were longer and higher in Ch. 1 than those in all other sites. These patterns are consistent with previously reports on the biomechanical role of the tongue in swallowing.

**Figure 10.**
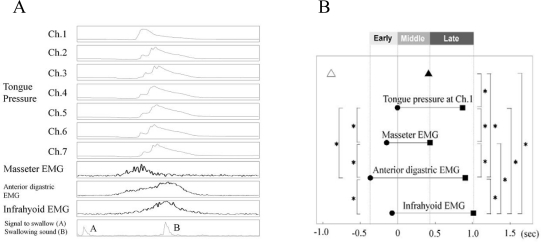
A: representative recording of tongue pressure at Chs. 1–7, integrated EMG of the masseter, submental, and infrahyoid muscles, signal to swallow, and swallowing sound, from which coordination of the tongue and oropharyngeal muscle activity was analyzed. B: coordination of tongue pressure produced at Ch. 1, activity of masseter, submental, and infrahyoid muscles, and swallowing sound during swallowing. The onset of tongue pressure at Ch. 1 was set to 0 seconds. ▵: signal to swallow; ▴: swallowing sound; •: onset; ▪: offset; * *P* < 0.05. Each timing of onset and offset was consistent with safe management of the bolus at the early, middle, and late stages of swallowing [48].

Measurement of tongue pressure production using this type of artificial palate with pressure sensors enabled analysis of temporal coordination patterns between the activity of the jaw and oropharyngeal muscles, and passage of the bolus during voluntarily triggered swallowing (Figure 10A). The temporal coordination patterns of the tongue and oropharyngeal muscles during voluntarily triggered swallow appeared to agree well with known safe bolus management (Figure 10B) [48].

**Figure 11.**
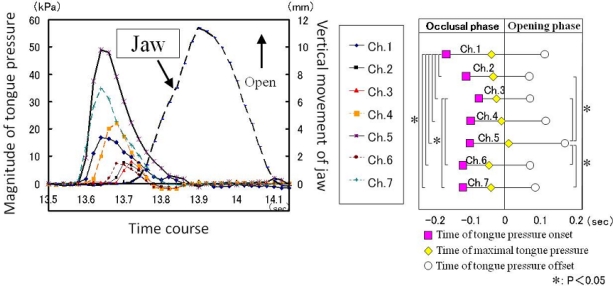
Representative recording of tongue pressure against the hard palate and the vertical trajectory of jaw movement in the masticatory cycle (left box), and coordination between tongue pressure and jaw movement (right box). Significant difference in sequential order is observed regarding the onset and offset of tongue pressure [49].

In combination with devices for measuring mandibular movement, the artificial palate is also an effective tool for assessing coordination between tongue pressure and jaw movement during mastication. Figure 11 shows the results of an experiment that confirmed a coordination pattern with mandibular movement, whereby tongue pressure develops in the occlusal phase and disappears in the opening phase during the masticatory cycle [49]. Furthermore, measurements obtained by the artificial palate clearly show that duration of tongue pressure during each masticatory cycle increased immediately prior to swallowing after fragmentation and bolus formation, along with an increase in the maximal magnitude of tongue pressure, revealing biomechanical changes of tongue movement that cannot be detected by imaging assessment alone [49]. This change quantitatively indicates activation of tongue movement associated with Stage II transport of gummy jelly bolus, as observed by VF (Figure 1D and Table 1).

### 4.4. Sensor Sheet

**Figure 12.**
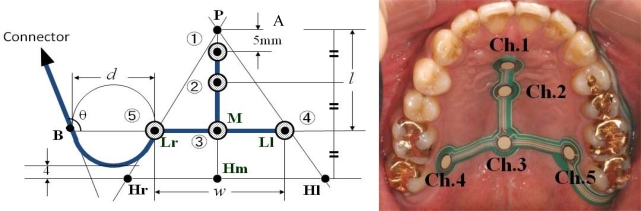
Design of the sensor sheet for measuring tongue pressure (A) and positioning of the sheet on the hard palate with denture adhesive (B) [52].

**Figure 13.**
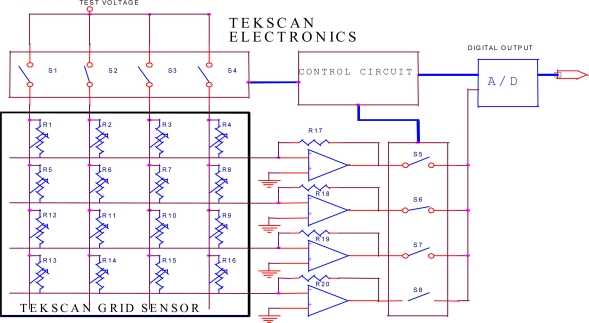
Structure of the I-Scan system [53].

The artificial palate is the most useful tool for quantitative analysis of basic tongue movement patterns in a natural mastication and swallowing setting because it has strictly standardized sensors and does not interrupt occlusal contact, which often occurs with probe insertion systems. However, this system has some drawbacks. It is difficult to apply to clinical practice because its production involves a highly advanced and costly technique. The palatal plates are fairly thick, so an adaption period is needed to overcome the discomfort felt in wearing them. Given these disadvantages, Hori *et al.*, [50–52] developed an easy-to-use sensor sheet for measuring tongue pressure. The ready-made sensor sheet is simply attached onto the palate in use and requires no acclimation time (Figure 12). The sensor sheet (an ultrathin sheet 0.1 mm thick with a rated capacity of 70 kPa, a measuring accuracy of 0.27 kPa and a sampling rate of 100 Hz) is connected to the general surface pressure distribution measurement system (I-scan, Nitta) and converts pressure to electrical signals via conducting ink in the sheet; the data are then recorded and displayed on a personal computer screen in real time (Figure 13) [54]. The electrical resistance of sensor cells under no load is almost infinite, while it decreases in inverse proportion to applied force. Each electrode reads changes in electric resistance values.

**Figure 14.**
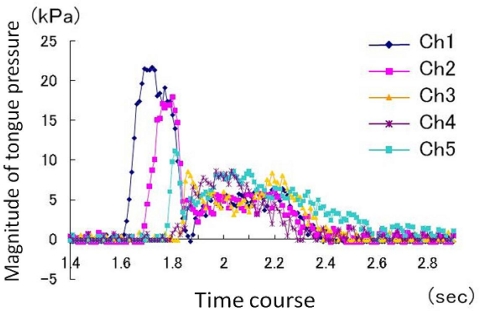
Representative recording of tongue pressure using a sensor sheet system during swallowing, in a healthy subject [52].

The T-shaped sensor sheet incorporates five pressure-sensing points. In use, the best fitting of three sizes can be chosen to suit the subject’s palate, which is then attached directly onto the palatal mucosa or the denture surface with a sheet-type denture adhesive. There is no standard setting of the pressure sensing points, as for an artificial palate, but a previous study confirmed that tongue pressure patterns (order of onset, duration, and maximal magnitude) can be adequately assessed at different palatal positions (Figure 14). Results of a clinical trial of a sensor sheet in 30 healthy subjects indicated that the anterio-median part of the hard palate (Ch. 1) headed a sequential tongue pressure production across channels and received the maximum pressure compared to other positions, which is in good agreement with the normal pattern of tongue pressure production recorded by an artificial palatal plate with pressure sensors [52]. The development of this tongue pressure sheet has enabled measurements to be obtained in a large number of subjects and the collection of clinical data in various diseases that present with symptoms of dysphagia. The usage of this sensor sheet is limited for research work at the present moment, but is expected to be commercially avairable in the near future.

**Figure 15.**
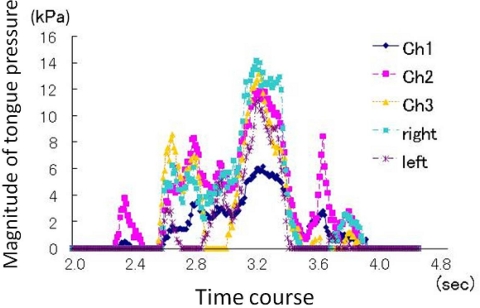
Tongue pressure production during swallowing water in a tongue cancer patient before glossectomy. Compared with a normal subject (see Figure 14), we observe a decline in the magnitude of tongue pressure in the anterio-median region (Ch. 1) and prolonged duration of tongue pressure.

Using this system, measurement of tongue pressure during swallowing reveals characteristic “disruption” of tongue pressure patterns in patients who present with symptoms of dysphagia related to different diseases. For example, in patients who received glossectomy, tongue pressure during swallowing was shown to have already decreased and have longer duration before surgery, and the relation of pressure magnitude on the midline of the palate to the pressure balance at the circumferential region was different from the patterns observed in healthy persons (Figure 15). In these patients, tongue pressure during swallowing showed a relationship with poor recovery after surgery. Maximal voluntary isometric contraction of the tongue was also decreased in these patients.

## 5. Sensing of Laryngeal Movement

### 5.1. Laryngeal Movement in Swallowing

The larynx is the entrance to the trachea as well as one of the vocal organs. In swallowing, it plays an important role in safely transporting the food bolus or liquid into the esophagus by rapidly moving the epiglottis to cut off the connection to the trachea, as if covering the trachea with a lid. This movement of the epiglottis is accomplished by moving the hyoid bone and the larynx in a coordinated manner. In the pharyngeal phase of swallowing, the hyoid bone is moved superiorly by activating the suprahyoid muscles after fixing the mandible in the closed position. Subsequently, the infrahyoid muscles are activated to move the larynx anterosuperiorly to approach the hyoid bone.

When assessing swallowing functions, laryngeal movement has to be understood and evaluated in the context of the entire swallowing process from the oral phase to the esophageal phase. Delayed or insufficient movement of the larynx impedes swallowing by allowing the food bolus or liquid to accidentally enter the trachea. We must therefore measure laryngeal movement including the motion of the epiglottis as accurately as possible, to evaluate the following parameters: (1) onset and completion of the swallowing process, particularly in the pharyngeal phase when the food bolus passes through the pharynx; and (2) the motion pattern and excursion of the larynx through the pharyngeal phase to the esophageal phase as well as its stability before and after movement. Such measurement should be carried out as non-invasively as possible.

Methods of sensing laryngeal movement can be classified roughly into three categories: (1) motion detection from the skin surface; (2) electrical impedance measurement; and (3) sagittal neck contour measurement, by which means we can evaluate vibration of the throat originating from laryngeal movement, electrical impedance of the neck, and changes in the sagittal contour of the throat, respectively. Recent Japanese advances in measurement technology for each category are described in this section.

### 5.2. Motion Detection from the Skin Surface

In swallowing, the larynx ascends and then descends, as mentioned in Section 2. This movement always causes skin movement or vibration in the anterior region of the neck over the larynx. Such skin movement has previously been measured using piezoelectric sensors [54], deviation transducers [55], and accelerometers [56] in an attempt to detect the onset of the laryngeal elevation or to assess overall motion of the larynx.

**Figure 16.**
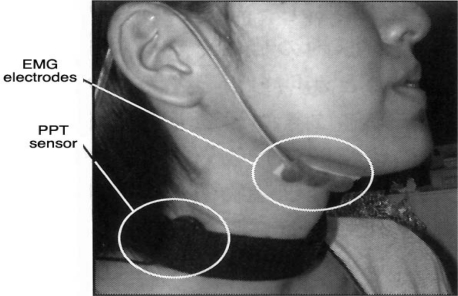
Piezo-Electric Pulse Transducer® and EMG electrodes mounted on the neck [59].

**Figure 17.**
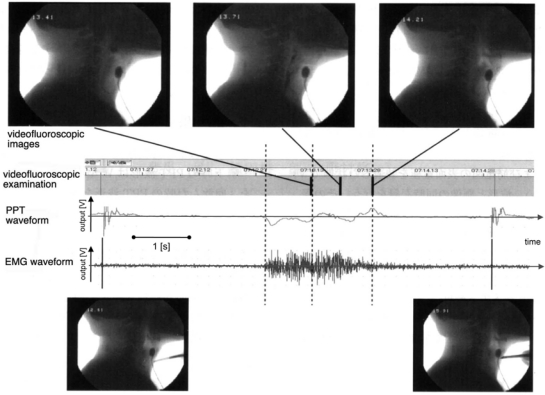
SVF images, PPT waveform, and electro-myographic data of suprahyoid muscles acquired simultaneously [59]. The measurements were synchronized by tapping the PPT with a metal rod before and after swallowing, as shown in the lower section of the figure.

Nomura and Toyosato recently applied a Piezo-Electric Pulse Transducer® (PPT; Model 1010, UFI Co., USA) to the detection of laryngeal movement [57–59]. This PPT has a frequency range of 2.5–5 kHz and was originally developed for recording peripheral pressure pulse. The PPT was used together with electromyographic measurement of the suprahyoid muscles. The transducer was mounted on the skin of the subject’s neck with a fixation belt, below the auricle and at the same level as the thyroid cartilage, to avoid detecting the pulse wave of the carotid arteries (Figure 16). To analyze the relationships among the pulse wave, electromyographic data, and actual movement of the larynx and food bolus, simultaneous measurements were performed using PPT, VF, and electromyography. Figure 17 demonstrates PPT and EMG waveforms and the three related VF images. The PPT wave started near the beginning of the pharyngeal phase with activation of the infrahyoid muscles.

The epiglottis moved shortly after the second wave appeared, and the PPT wave disappeared in the esophageal phase. Because the PPT waveform in an individual patient is highly reproducible under the same swallowing conditions, the above findings suggest that the times of food-bolus passage and epiglottis movement could be estimated from the PPT waveforms. This method has the advantage of being applicable at the patient’s bedside, and also has the potential to be used for automatically counting the frequency of swallowing in the repetitive saliva swallowing test (RSST) and optimizing the patient’s head position during chewing to avoid aspiration. The mechanism of PPT-waveform generation during swallowing, however, has yet to be clarified.

### 5.3. Electric Impedance Measurement

**Figure 18.**
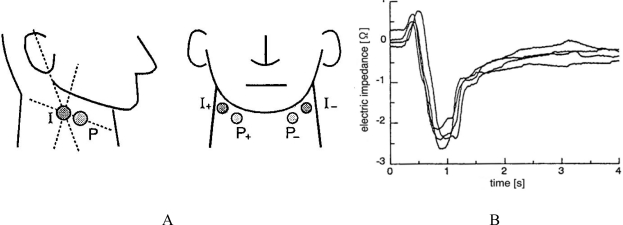
Impedance pharyngography [60]. A: position of the four electrodes, denoted as I_+_, P_+_, I_−_ and P_−_, attached to the neck skin surface. B: change in electric impedance over time during swallowing.

Laryngeal movement can be detected indirectly by measuring physiochemical changes in the neck. Using this detection method, Yamamoto *et al.*, developed impedance pharyngography (IPG) [60,61], which is an electrical impedance-based method of measuring swallowing activity. As shown in Figure 18A, two electrode pairs, denoted as (I_+_, P_+_) and (I_–_, P_–_), are attached to the neck skin surface to avoid skin-impedance effects [60]. Electrode I_+_ provides the neck with an alternating electric current of 0.5 mA and 50 kHz determined empirically to maximize sensitivity, while electrode I_–_ is grounded. Voltage data detected by electrodes P_+_ and P_–_ are amplified in a differential manner. The output envelope representing electric impedance is then obtained using a combination of a synchronous detector and a low-pass filter. Figure 18B demonstrates several waveforms of impedance pharyngographs, where electric impedance changed in the range of a few ohms over time when 10 cc of water was swallowed; this was caused by the changing shape and position of the pharynx as well as the effects of the water. This method has the advantage of being applicable to bedside examinations; however, the IPG waveform varies significantly among subjects, probably due to morphological differences, necessitating quantification of the waveform in a general manner for comparison. The relationship between the change in electrical impedance and movement of the pharynx and food bolus, however, remains unclear, preventing the application of IPG to detailed analysis of swallowing functions.

### 5.4. Sagittal Neck-Silhouette Measurement

Waveforms provided by the measurement methods described above enable indirect estimation of laryngeal movement in swallowing, but their clinical application is restricted because the relationship between the waveforms has yet to be established. In an attempt to eliminate ambiguity in understanding laryngeal movement from the waveforms, Hayashi *et al.*, are continuing to develop a swallowing-function evaluation system (*SFN*) capable of simultaneously measuring movement of the skin surface in the anterior neck, electromyographic activity of the suprahyoid muscles, and swallowing sounds [62–64]. This section describes the principles of detecting laryngeal movement using the *SFN* series.

**Figure 19.**
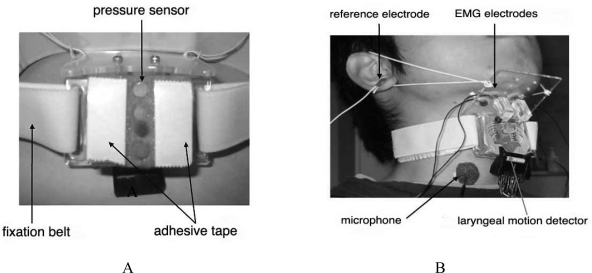
Swallowing-function evaluation system, *SFN*-1 [62], capable of measuring laryngeal movement, EMG activity of suprahyoid muscles, and swallowing sounds. A: sensing surface of laryngeal movement detector. B: sensors mounted on the anterior region of the neck.

**Figure 20.**
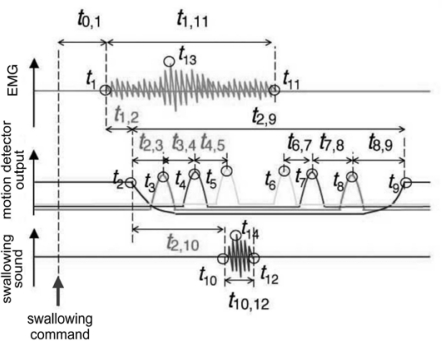
Analysis parameters used in *SFN*-1 [62]: Times {*t_i_*: *i* = 1,2,...,14} of characteristic points in waveforms and inter-point interval {*t_i,j_*: *i*, *j* = 1,2,...,12}.

In the first version of the device, *SFN*-1, a motion detector with four aligned pressure sensors (Figure 19A) was mounted on the anterior region of the neck (Figure 19B) to record the ascent and descent of the thyroid cartilage. Sensor output reaches a maximum when the cartilage passes just below it, enabling us to estimate laryngeal movement from the peak times in the sensor-output waveforms, as illustrated in Figure 19. Because the detector was mounted so that the lowest sensor was located over the cartilage at rest, the output of the lowest sensor decreased first during swallowing, followed by sequential peaks in other sensor-output waveforms, as illustrated in the middle section of Figure 20. Using this measurement principle, the motion detector is applicable primarily to male subjects because of the prominence of their larynx. EMG activity of the suprahyoid muscles and swallowing sounds were also recorded simultaneously, together with laryngeal movement. The times at which characteristic points in all waveforms occurred were then measured, and are denoted as *t_i_* (*i* = 1, 2,...,14) in Figure 20. The time intervals between these points, denoted as *t_i,j_* (*i*, *j* = 1, 2,...,12), were then computed. The onset time (*t*_1_) of suprahyoid muscle activities is known to almost coincide with the beginning of the oral phase of swallowing. Results of preliminary experiments with simultaneous measurement of *SFN* and videofluoroscopic imaging demonstrated that the trachea was closed by movement of the epiglottis at close to time *t*_4_, when the thyroid cartilage was just below the third pressure sensor (located 16 mm distant from the first). Hence, delay in the onset of laryngeal movement could lengthen the time interval *t*_1,2_, while delay of tracheal closure could lengthen time intervals *t*_1,4_ and *t*_2,4_. The speed of thyroid cartilage motion could be estimated from the time intervals *t*_2,3_, *t*_3,4_, and *t*_4,5_. Such abundant information regarding the subject’s swallowing functions cannot be obtained from individual non-invasive measurements of larynx motion, EMG activity, and swallowing sound. Swallowing experiments using rice gruel demonstrated that food texture had the greatest effect on the initial speed parameter *t*_2,3_ [62], supporting the idea that the initial stage of the pharyngeal phase before epiglottal movement could be modified significantly by food texture.

**Figure 21.**
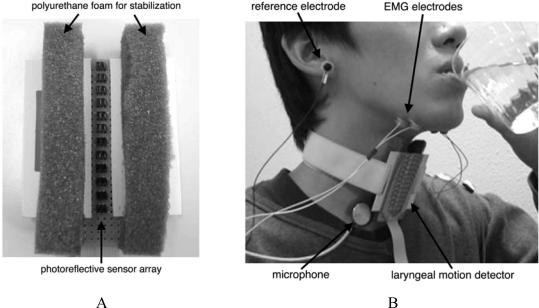
Laryngeal motion detector *SFN*-2 [63,64] incorporating a photo-reflective sensor. A: sensing surface of the detector. B: various sensors mounted on the anterior region of the neck.

Although the first version, *SFN*-1, could measure several features of swallowing, it had two defects: because the detector was attached directly and firmly to the anterior region of the neck, it exerted considerable pressure in this region, and it was unable to detect continuous movement of the larynx. In an attempt to eliminate these problems, *SFN*-1 was completely revised to create the second-generation model, *SFN*-2, in which the sensing device was updated from a pressure sensor to a photo-reflective sensor [63,64]. This modification enabled continuous detection of laryngeal movement in male subjects without exerting pressure on the anterior region of the neck. The photo-reflective sensor is an electronic device combining a light emitting diode (LED) and a photo-diode. It can detect the intensity of reflected light in inverse proportion to the square of the distance between the LED and the opposing surface. This device was applied to non-contact measurement of the skin surface contour in the anterior region of the neck, as shown in Figure 21.

**Figure 22.**
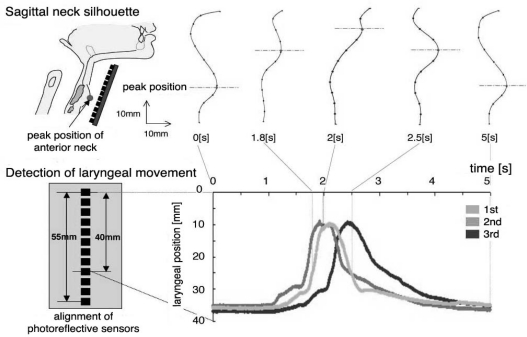
Sagittal neck-silhouette measurement using the laryngeal movement detector of *SFN*-2 [63,64] and automatic detection of laryngeal movement. Laryngeal position was defined as the position of the peak of the skin surface, and could be tracked automatically. Three trajectories of the same subject are depicted to demonstrate waveform reproducibility.

Twelve photo-reflective sensors were aligned on the active surface of the laryngeal motion detector at intervals of 5 mm (Figure 21A), and this detector was mounted on the anterior region of the neck using a fixation belt (Figure 21B). The position of the motion detector was stabilized using polyurethane foam in contact with the skin surface of the neck (Figure 21A), while the sensors were located above the skin surface to avoid interference with laryngeal movement. The detector provides distance data {*d_i_: i* = 1, 2,...,12} obtained from the sensors. The measurement error of the distance was verified to be 0.05 ± 0.03 mm on average, while its resolution was verified to be 0.16 mm (accuracy measured by root mean square error using white drafting paper as a reflective surface) [63]. By interpolating the distance data obtained at a rate of 400 Hz, time-series data of the sagittal contour of the neck can be produced at intervals of 0.55 mm, as shown in the upper section of Figure 22. The vertical position of the larynx was defined as that of the most anterior position observed in the contour, which could be tracked automatically.

The lower section of Figure 22 shows three laryngeal trajectories in the same subject, depicted as time-series data of the vertical position of the larynx. Each trajectory can be divided roughly into three different phases, as shown in Figure 22. In the first phase, the larynx began to ascend relatively slowly from the rest position for a distance of 5–7 mm, then ascended rapidly in the second phase when swallowing had taken place. The larynx finally returned slowly to the initial position in the esophageal phase of swallowing. Such features of laryngeal movement agreed well with those obtained from VF observations. Preliminary experiments demonstrated that the waveform of the laryngeal trajectory of elderly people with dysphasia appeared to differ from that of healthy people in three respects: (1) the rest position was instable; (2) the first phase was extended in both duration and amplitude; and (3) the second phase was extended in duration and/or divided into two subsections because ascension of the larynx slowed down or even stalled. Quantification of these phenomena is a task for a future study.

**Figure 23.**
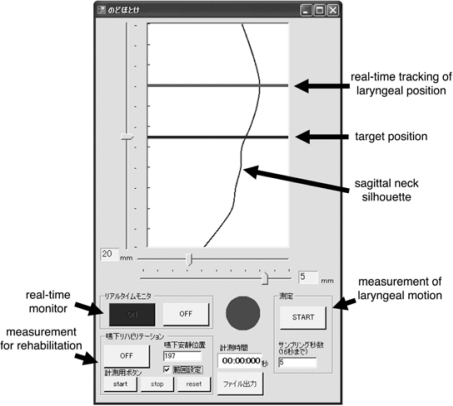
Visual feedback subsystem of the *SFN*-2 for application of laryngeal elevation exercises to patients with dysphagia. This subsystem provides a sagittal neck silhouette and laryngeal position on the computer screen in real time.

A visual feedback system was recently incorporated into *SFN*-2, enabling patients’ sagittal neck contour and laryngeal position to be visualized on a computer screen in real time, as shown in Figure 23. This modification was made so that the *SFN*-2 could be applied to swallowing rehabilitation in the clinical setting. It is not easy for patients with severe dysphagia to intentionally elevate their larynx for a short period of time during laryngeal elevation exercises, even when its position is checked manually. To help patients to perform such exercises, a visual feedback system was designed to display patients’ laryngeal position in real time as well as in the target position (Figure 23). Preliminary experiments after using the visual feedback system with healthy male volunteers showed a significant increase in the highest laryngeal position subjects were instructed to maintain by themselves for 10 seconds. Clinical tests employing patients with dysphagia should be performed in the future to establish an *SFN*-2-based rehabilitation procedure as well as to evaluate its rehabilitation effects.

## 6. Conclusions

Sensing systems developed for monitoring the movement of the lips, tongue, and larynx were presented in this review. All these systems can quantitatively assess the movement of different organs using simple, non-invasive methods. In Japan, the research and development of sensing systems for oropharyngeal swallowing movement are flourishing. In stark contrast to the USA, where dysphagia rehabilitation is generally left to speech therapists, the dentistry community in Japan has shown great interest in oromaxillary movement analysis and a commitment to dysphagia rehabilitation.

No sensing technique introduced in this review can be used to evaluate the swallowing function in its entirety, though these methods are much more user-friendly, quantitative, and practicable compared with past techniques. Future development of an integrated system in which data obtained by each technique is synchronized will realize the non-invasive and quantitative evaluation of the whole swallowing function in the clinical state. Application of such an evaluation system to dysphagic patients with various diseases is also a subject for future investigation. As well as accumulating more clinical data, the sensitivity and specificity for dysphagia should be established to diagnose and evaluate the disease based on measured parameters. Quantitative assessment of the swallowing function would make diagnosis, treatment, and rehabilitation of dysphagia more efficient, if established. However, the contribution of these sensing techniques would extend to include the development of treatment for diseases that are clinically indicated by dysphagia, a reduction in the social and medical costs of caring for dysphagia patients, and the development of safer food. Further technical contributions are required from engineering fields.

## Figures and Tables

**Figure 2.**
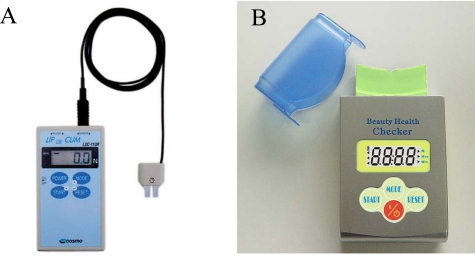
Commercially available lip-force measuring devices. A: LIP DE CUM (left). B: Beauty Health Checker (right). (Photos courtesy of Cosmo Instruments and Patakara).

**Table 1.** Outline of tongue movement described in the Process Model [7].

**Ingestion**
The mouth opens widely as the tongue drops down to make room in the mouth for the bite of food to enter.
**Stage I transport**
The tongue moves posteriorly in the oral cavity, carrying the bite of food on its surface, and rotates to deliver the food to the chewing surface of the molars.
**Food processing**
Once food reaches the molar region, it is softened and reduced in size as it is chewed, manipulated, and mixed with saliva. During chewing, jaw movement is linked to cyclic motions of the tongue and hyoid bone. The tongue moves upward and downward in association with jaw closing and opening, and also forward and backward. These horizontal motions of the tongue are out of phase with its vertical motions.
**Stage II transport**
The portion of food bolus is propelled through the faucial pillars into the oropharynx where it is stored in anticipation of swallowing. Some food remains in the oral cavity, where processing continues. During Stage II transport, cyclic motions of the jaw and tongue continue in a linked pattern. In some cycles, food is pulled backward on the surface of the tongue, while in other cycles the tongue pushes it against the palate and squeezes it back along the palate and into the oropharynx.
**Hypopharyngeal transit**
As the pharyngeal swallow begins, the food bolus in the oropharynx is propelled through the hypopharynx into the esophagus. Bolus propulsion is produced primarily by backward thrust of the tongue onto the oropharynx.
